# Data-Independent Acquisition Proteomics Reveals the Effects of Red and Blue Light on the Growth and Development of Moso Bamboo (*Phyllostachys edulis*) Seedlings

**DOI:** 10.3390/ijms24065103

**Published:** 2023-03-07

**Authors:** Ke Li, Luyao Ji, Yaoyun Xing, Zecheng Zuo, Li Zhang

**Affiliations:** 1 Basic Forestry and Proteomics Research Center, Fujian Agriculture and Forestry University, Fuzhou 350002, China; 2 Jilin Province Engineering Laboratory of Plant Genetic Improvement, College of Plant Science, Jilin University, Changchun 130062, China

**Keywords:** moso bamboo, red light, blue light, data-independent acquisition proteomics, growth and development

## Abstract

Moso bamboo is a rapidly growing species with significant economic, social, and cultural value. Transplanting moso bamboo container seedlings for afforestation has become a cost-effective method. The growth and development of the seedlings is greatly affected by the quality of light, including light morphogenesis, photosynthesis, and secondary metabolite production. Therefore, studies on the effects of specific light wavelengths on the physiology and proteome of moso bamboo seedlings are crucial. In this study, moso bamboo seedlings were germinated in darkness and then exposed to blue and red light conditions for 14 days. The effects of these light treatments on seedling growth and development were observed and compared through proteomics analysis. Results showed that moso bamboo has higher chlorophyll content and photosynthetic efficiency under blue light, while it displays longer internode and root length, more dry weight, and higher cellulose content under red light. Proteomics analysis reveals that these changes under red light are likely caused by the increased content of cellulase CSEA, specifically expressed cell wall synthetic proteins, and up-regulated auxin transporter ABCB19 in red light. Additionally, blue light is found to promote the expression of proteins constituting photosystem II, such as PsbP and PsbQ, more than red light. These findings provide new insights into the growth and development of moso bamboo seedlings regulated by different light qualities.

## 1. Introduction

Bamboo is a group of perennial herbaceous plants in the Poaceae family, grown in a wide range of climates and widespread in the Asia-Pacific, the Americas and Africa [1]. Except for its rapid growth [2], moso bamboo is also specific in terms of its once-in-a-lifetime flowering and a long vegetative phase of 60 years or more before flowering [3], a unique rhizome-dependent propagation system [4], adult stems of 20 cm in diameter with high mechanical strength [1], etc., resulting in various commercial applications of moso bamboo in the food and pharmaceutical industries, raw wood processing, and other fields such as household supplies, housing construction and decoration, and renewable energy (charcoal and biofuels) [1,5,6,7]. Afforestation is a necessary way to expand the production of moso bamboo. The traditional way of afforestation is to transplant the mother bamboo; however, the survival rate is very low and extensive labor is required. An alternative is to cultivate moso bamboo container seedlings, which saves labor costs and increases survival rates [8]. It is widely known that light plays a vital role in the growth and development of plant seedlings [9]. So, inevitably, the cultivation of moso bamboo container seedlings requires strict control of light. In addition, moso bamboo grown in total shade will not survive; although the mother plant provides some carbohydrates to the moso bamboo seedlings, this indicates the necessity of light for the growth of moso bamboo seedlings [10].

Light signals are involved in the regulation of plant growth, development and metabolism through light quality, light intensity and photoperiod. Red light (600–700 nm) and blue light (400–500 nm) have been extensively studied as the main light qualities used by plants for photosynthesis [11]. Plants have also evolved photoreceptors, such as phytochromes [12], cryptochromes [13], phototropins and the Zeitlupe (ZTL)/Flavin-binding Kelch F-box1 (FKF1)/LOV Kelch protein 2 (LKP2) family to transduce red and blue light signals [14,15]. Furthermore, photoreceptors control plant growth and development in different ways [16], making it critical to investigate the effects of monochromatic red and blue light on the growing process in plants. The physiological phenotypes of plants growing in red light and blue light are significantly different, mainly in morphogenesis, photosynthetic performance and secondary metabolites [17]. For example, the hypocotyls of *Arabidopsis* and tomato grown under red light are longer than those grown under blue light [18]; the chlorophyll content in leaves of rice seedlings grown under red light is lower than that under blue light [19]. The regulation of red and blue light for plant growth and development also differs between species: for example, the hypocotyls of cucumber grown under blue light are longer than those under red light [20]; the chlorophyll concentration in leaves from chrysanthemum grown under red and blue light is not significantly different [21]. However, the effects of different light qualities on the growth of moso bamboo seedlings have not been reported.

Although a genetic transformation system for moso bamboo has recently been established [22], the long vegetative phase of moso bamboo makes it difficult to obtain transgenic seeds, leading to difficulty in applying traditional genetic methods to study the molecular mechanisms of photomorphogenesis in moso bamboo seedlings after seed germination. Therefore, omics has become an effective method for studying the potential molecular mechanisms underlying the effects of different light qualities on the growth of bamboo. Proteins are considered to be the biological macromolecules closest to “gene function” and directly related to phenotype [23]. Therefore, proteomics has become a common and important tool to reveal functional proteins in plant development and metabolic processes, especially for non-model organisms [24]. Quantitative proteomics technology based on data-independent acquisition (DIA) is a technique that has been used in recent years for large scale protein quantification and identification. In contrast to data-dependent acquisition (DDA), DIA proteomic analysis has been increasingly used in various proteomic studies due to its broad protein coverage, high reproducibility and accuracy [25]. To investigate the effects of red light and blue light on the growth and development of moso bamboo seedlings and identify possible molecular mechanisms underlying these effects, here, we compared the morphological and developmental characteristics of moso bamboo seedlings grown for 14 days under monochromatic red light, monochromatic blue light and darkness, and analyzed the proteomic characteristics of these moso bamboo seedlings using DIA-MS analysis. The results showed that blue light motivates chlorophyll synthesis and photosynthetic photochemical properties in moso bamboo and promotes the expression of photosystem II constituent proteins, such as PsbP and PsbQ. On the other hand, red light mainly increases the accumulation of sucrose, fructose and cellulose in moso bamboo and up-regulates cell wall synthesis proteins. Moso bamboo grown under red light also exhibits higher dry weight and longer stem and root lengths. These findings provide a theoretical basis to understand the protein regulatory network and to determine protein functions that regulate the growth and development of moso bamboo seedlings under red and blue light, and provides new insights for the future application of genetic engineering in this species and the regulation of plant seedling growth by red and blue light.

## 2. Results

### 2.1. Effects of Red and Blue Light on the Photomorphogenesis of Moso Bamboo Seedlings

**Figure 1:**
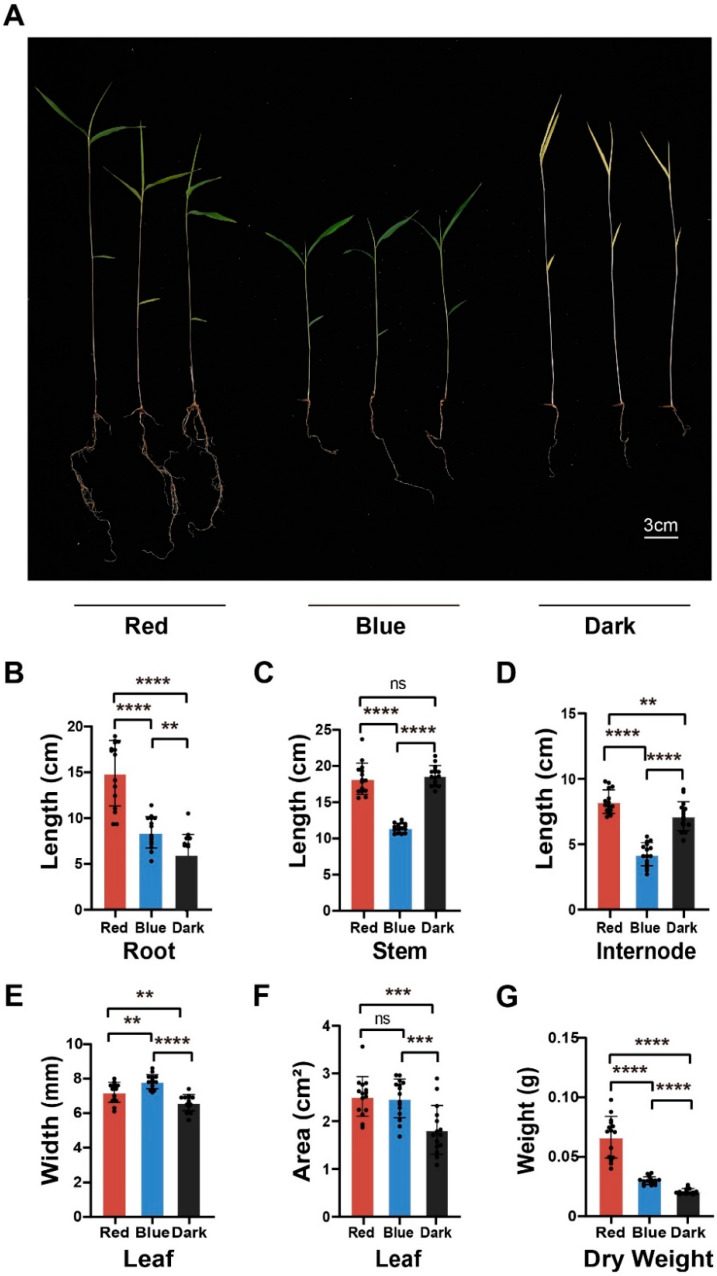
Effects of red and blue light treatments on the growth and development of moso bamboo seedlings. (**A**) The representative phenotypic image of moso bamboo seedlings under different light treatments. After the seeds germinated in the dark, they were transferred to red light (650 nm), blue light (450 nm) and dark conditions for 14 days. Scale bar = 3 cm. (**B**–**G**) Quantification of the root length (the length of the primary root) (**B**), stem length (**C**), internode length (the length between the second and third leaf) (**D**), leaf width (the width of the widest position of the second leaf) (**E**), leaf area (the area of the second leaf) (**F**), dry weight (**G**) of the bamboo seedlings treated with different light conditions. Data are presented as mean ± SD (*n* = 15 seedlings). Significant differences were analyzed by two-tailed Student’s *t*-test (^ns^ *p* > 0.05, ** *p* < 0.01, *** *p* < 0.001, **** *p* < 0.0001).

After seed germination in the natural environment, seedlings are exposed to light and enter the process of photomorphogenesis [26]. As the natural light consists of various light qualities, it is difficult to observe the effect of monochromatic light on photomorphogenesis. To investigate the effect of monochromatic red and blue light on moso bamboo photomorphogenesis, we transferred the moso bamboo germinating in the dark to red light, blue light or the original darkness for 14 days (Figure 1A). Moso bamboo grown in darkness underwent etiolation and limited primary root growth (Figure 1A), while the seedlings grown under red light showed a significant increase in root length (Figure 1B) and lateral root growth. Moreover, the stem length of moso bamboo grown under red light was significantly longer than that under blue light, but there was no significant difference in stem length between red light and darkness (Figure 1C). In addition, the stem diameter of seedlings grown under red light was significantly larger than that of seedlings grown in darkness. Interestingly, there was no significant difference in stem diameter between darkness and blue light conditions (Figure S1A). These results indicate that, in contrast to blue light, red light not only promotes longitudinal elongation but also supports the horizontal growth of young moso bamboo seedlings. Moreover, the internode length under red light (Figure 1D) was significantly longer than that under blue light and darkness, and the internode length under darkness was also significantly longer than that under blue light, indicating that the stem length of moso bamboo under red light is mainly attributed to the elongation of internodes. The first elongated internode under darkness (Figure S1B) was significantly longer than that under red and blue light, and the first elongated internode under red light was also significantly longer than that under blue light, suggesting that the stem length of moso bamboo under darkness is mainly reflected in the elongation of the first internode. Leaf width and area determine the efficiency of photosynthesis in plants. Our findings showed that the leaf width of moso bamboo under red and blue light (Figure 1E) was significantly broader than that in the dark, and the leaf width under blue light was significantly broader than that under red light. However, there was no difference in leaf area under red and blue light (Figure 1F). We also found that the dry weight of moso bamboo seedlings under red light (Figure 1G) was significantly higher than that under blue light and darkness, and the dry weight under blue light was significantly higher than that under darkness. This suggests that red light is more effective than blue light in promoting the accumulation of organic matter in moso bamboo seedlings. In conclusion, our results indicate that red and blue light have distinct effects on the photomorphogenesis of moso bamboo seedlings.

### 2.2. Quantitative Proteomics Analysis

To gain more insight into the effects of red and blue light on the growth and development of moso bamboo seedlings, we compared and analyzed the proteomes of moso bamboo seedlings grown under red and blue light for 14 days using the DIA mass spectrometry identification technique. Then, we analyzed the DIA data obtained using the directDIA module in the Spectronaut 16 software (Biognosys AG, Schlieren, Switzerland).

**Figure 2:**
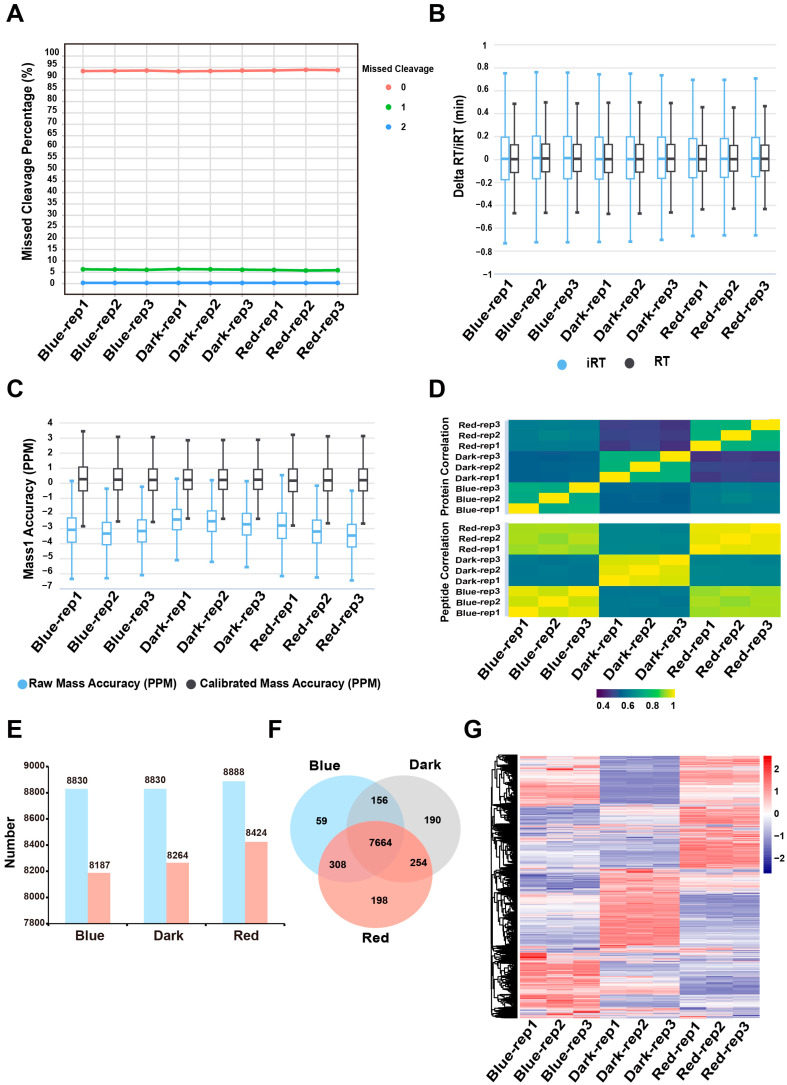
Quantitative proteomic analysis. (**A**) The percentage of peptides containing one (green dots), two (blue dots) or no missed cleavages (red dots) in each sample. The “rep” represents “replication”, the same as below. (**B**) The delta RT/iRT is the difference between a peptide theoretical and empirical RT/iRT, computed from the linear iRT/RT regression function [27]. (**C**) The plot of mass accuracy before and after calibration. These plots display the mass accuracy for all identified peptides at the precursor (MS1) level. The mass accuracy is calculated by dividing the (observed *m*/*z*-theoretical *m*/*z*) by the theoretical *m*/*z* (in ppm). (**D**) The sample correlation matrix shows correlation of peptide/protein group quantities between all samples. (**E**) The numbers of identified proteins and quantified proteins in the moso bamboo samples under blue light, dark light and red light conditions. The blue bar represents the number of identified proteins. The red bar represents the number of quantified proteins (the proteins identified by all three replicates). (**F**) Venn diagram showing the specific quantified and shared quantified proteins of the moso bamboo seedlings under different light conditions. (**G**) Heat map of expression profiles for the shared quantified proteins in moso bamboo seedlings under different light conditions.

A quality control (QC) analysis was performed to assess the quality of the proteomics data. Our results showed that the enzymatic digestion efficiency of each sample was high, with nearly 95% of the peptides having no missed cleavages, and only a small percentage (5%) having one missed cleavage (Figure 2A). The delta RT/iRT values were in the range of ±0.5 min/±0.8 min for each sample, indicating that the actual retention times of the peptides in the liquid phase did not differ significantly from the theoretical retention and the samples had good reproducibility (Figure 2B). Additionally, the mass accuracy of each sample before and after calibration was within 5 ppm between different replicates of the same treatment, indicating a relatively stable mass spectrum with good sample reproducibility (Figure 2C). Next, we compared the Pearson correlation coefficients between the number of peptide groups and protein groups of the different samples. The results showed a low correlation between samples from different treatments and a high correlation between samples from different replicates of the same treatment (Figure 2D). Further analysis of the Spectronaut quantification results showed that a total of 9033 moso bamboo proteins were identified. The maximum number of proteins identified under red light treatment was 8888, and 8830 proteins were identified under both dark and blue light. We defined proteins identified in all replicates under one type of light treatment as the proteins quantified under that treatment. The results showed that there were 8187 proteins quantified under blue light, 8264 proteins quantified in the dark and 8424 proteins quantified under red light (Figure 2E, Table S1). A total of 7664 proteins were co-quantified under all three light conditions (Figure 2F). Further analysis using principal component analysis (PCA) on these 7664 proteins revealed that samples from the three different light treatments were well separated and samples from the same treatment were well clustered (Figure S2). Additionally, a heat map of protein abundance values also showed a large difference in protein abundance between samples from different treatments, but a small difference between samples from the same treatment (Figure 2G). These analyses indicated that the proteomics data from this study are of good reproducibility and reliable quality, with significant differences in protein abundance between samples with different light treatments.

### 2.3. Functional Analysis of the Specific Quantitative Proteins of Moso Bamboo under Red and Blue Light

**Figure 3:**
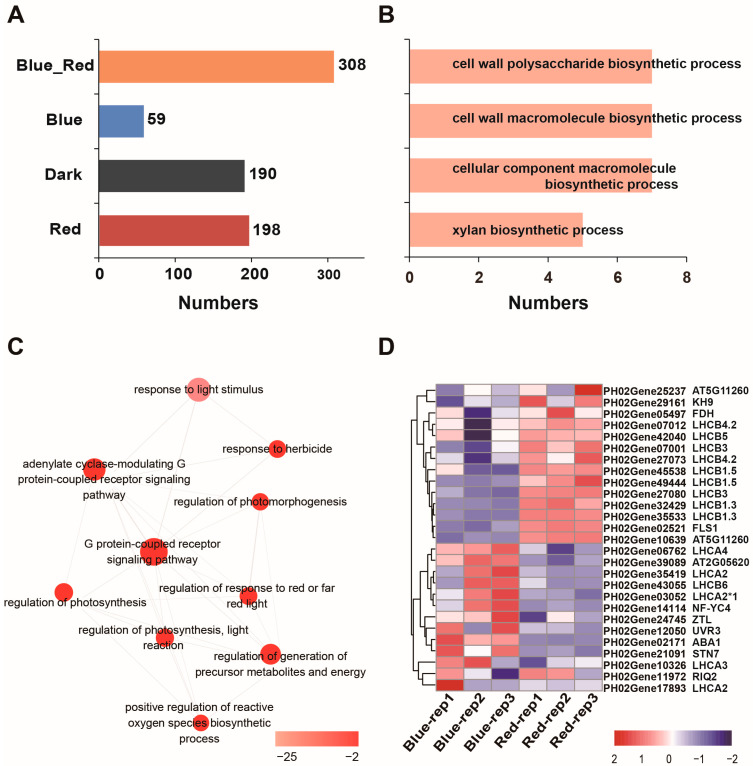
Gene ontology analyses of moso bamboo proteins under red light and blue light conditions. (**A**) The number of specific quantified proteins in moso bamboo under different light conditions. Blue_Red represents the group of proteins identified under red and blue light, but not in the dark. (**B**) Gene ontology (GO) enrichment analysis of the red light-specific quantified proteins. (**C**) Interactive graph of the gene ontology enrichment analysis of the proteins in Blue_Red in (**A**). The color of the bubble corresponds to the Log10 (FDR value of the GO term). The size of the bubble corresponds to the Log10 (number of annotations for the GO term ID in selected species in the EBI GOA database). (**D**) The heatmap showing the expression levels of specific quantified light stimulated proteins in Blue-Red.

We statistically compared the results of protein quantification in moso bamboo seedlings under different light conditions and found that 308 proteins were quantified in both blue and red light, but not in darkness, and we defined these proteins as Blue_Red-specific proteins. Fifty-nine proteins were quantified specifically under blue light, 190 proteins were quantified specifically in the dark, and 198 proteins were quantified specifically under red light (Figure 3A). To explore the function of moso bamboo specific quantified proteins under different light conditions, we performed gene ontology (GO) analysis on the specific quantified protein data under different light conditions. Dark and blue light-specific quantified proteins were not enriched for any protein functionS, whereas red light-specific quantified proteins were mainly involved in “cell wall polysaccharide biosynthetic process”, “cell wall macromolecule biosynthetic process”, “cell component macromolecule biosynthetic process” and “xylan biosynthetic process” (Figure 3B). These included the IRX (IRREGULAR XYLEM) proteins (PH02Gene00471, PH02Gene00093 and PH02Gene07283) involved in plant secondary wall synthesis and the GMX2 (GLUCURONOXYLAN METHYLTRANSFERASE 2) protein (PH02Gene37597) (Table S2). Next, we found that Blue_Red-specific proteins were mainly involved in biological processes such as “response to light stimulus”, “G protein-coupled receptor signaling pathway”, “regulation of photomorphogenesis”, “regulation of photosynthesis” and “regulation of response to red or far red light” (Figure 3C). GO analysis revealed that 27 blue and red light-specific quantification proteins were identified as a function of “response to light stimulus” and the overall difference in protein abundance between blue and red light treated samples was significant, as evidenced by the fact that 14 proteins were expressed at higher levels in red light than in blue light, including nine LHCB proteins (LIGHT-HARVESTING CHLOROPHYLL B-BINDING PROTEIN) (PH02Gene07012, PH02Gene42040, PH02Gene07001, PH02Gene27073, PH02Gene27080, PH02Gene45538, PH02Gene49444, PH02Gene32429, PH02Gene35533). There were 13 proteins with higher protein abundance under blue light than under red light, including five LHCA proteins (LIGHT-HARVESTING CHLOROPHYLL A-BINDING PROTEIN) (PH02Gene06762, PH02Gene35419, PH02Gene03052, PH02Gene10326, PH02Gene17893), one LHCB6 protein (PH02Gene43055) and the blue light receptor ZTL (PH02Gene24745) (Figure 3D). At the same time, we analyzed PH02Gene25237 and PH02Gene10639 in the “regulation of photomorphogenesis” and found that both genes were homologs of *Arabidopsis* HY5 (ELONGATED HYPOCOTYL 5) and red light promoted the expression of PH02Gene10639 (Figure S3). The above results indicate that moso bamboo specifically expresses some proteins related to cell wall synthesis in red light. Although both red and blue light specifically promote the expression of chlorophyll-binding proteins compared to darkness, they function quite differently: red light promotes the expression of LHCB and blue light promotes the expression of LHCA more.

### 2.4. Functional Analysis of Differentially Expressed Proteins in Moso Bamboo under Blue and Red Light

**Figure 4:**
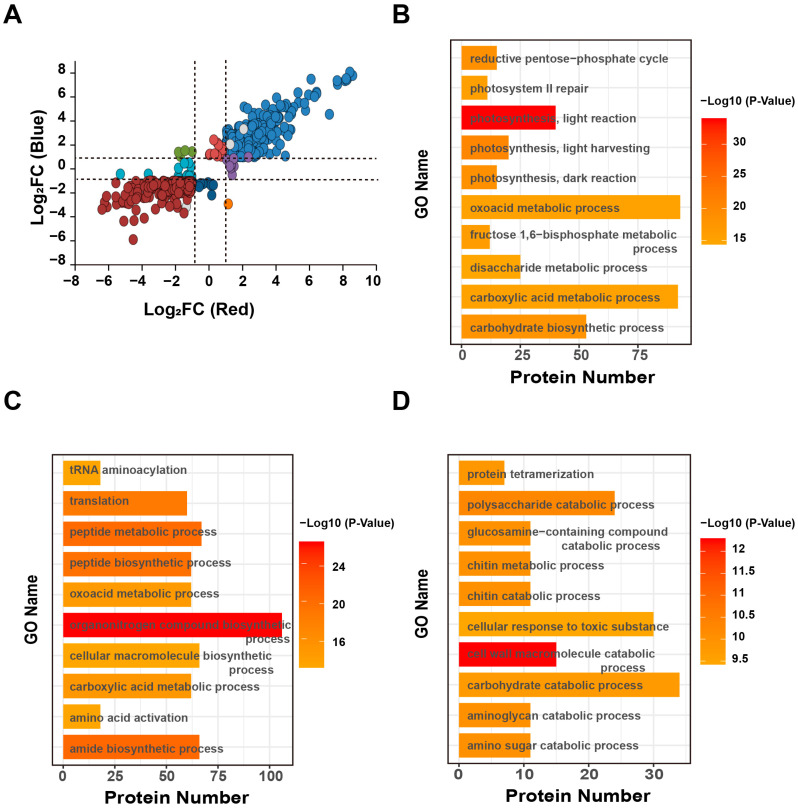
Differences in protein expression in moso bamboo under red and blue light treatments. (**A**) Scatter plot showing the association between the blue light-responsive proteins and the red light-responsive proteins in moso bamboo. The dashed lines indicate Log2 (FC) = ±1. The steel blue and dark red plots indicated the co-upregulated/downregulated proteins under red and blue light treatments. Green and orange plots indicated the proteins regulated in the opposite direction between red and blue light treatments. Red and navy plots indicated the proteins upregulated/downregulated under blue light and unchanged under red light. Purple and cyan plots indicated the proteins upregulated/downregulated under red light and unchanged under blue light. Gray plots indicated the proteins with *p* value > 0.01. (**B**) GO enrichment analysis of the blue and red light co-responsive proteins shown in (**A**). (**C**) GO enrichment analysis of the blue light-specific responsive proteins shown in (**A**). (**D**) GO enrichment analysis of the red light-specific responsive proteins shown in (**A**).

A total of 7664 moso bamboo proteins were co-quantified in dark, red and blue light conditions (Figure 2F). To further investigate the effects of light on protein expression, we then used the abundance of moso bamboo proteins in the dark as a control and established a cutoff of a fold-change ratio (Blue/Dark or Red/Dark) > 2 or <0.5 and a *p*-value < 0.01 to define differentially expressed proteins (DEPs, light-responsive proteins) in moso bamboo. Finally, we obtained 1508 blue light-responsive proteins and 1867 red light-responsive proteins, and then divided these light-responsive proteins into three groups: (1) 984 proteins were found to be co-regulated by both blue and red light. Of these, 99.39% were up- or down-regulated by both light conditions, with 793 proteins up-regulated and 185 proteins down-regulated. Only five proteins were up-regulated by blue light and down-regulated by red light, and one protein was down-regulated by blue light and up-regulated by red light; (2) 524 proteins were specifically regulated by blue light, most of which (72%) were up-regulated (378 proteins specifically up-regulated by blue light and 146 proteins specifically down-regulated by blue light); (3) 883 proteins were specifically regulated by red light, including 432 red light-specific up-regulated proteins and 451 red light-specific down-regulated proteins (Figure 4A and Figure S4A). To better understand the functions of these light-responsive proteins, we performed GO analysis on the above three groups of light-responsive proteins. The results showed that the GO terms related to photosynthesis, including “photosystem II repair”, “photosynthesis, light reaction”, “photosynthesis, light harvesting”, and “photosynthesis, dark reaction”, were enriched in proteins co-regulated by blue and red light (Figure 4B). GO terms related to protein synthesis and degradation, including “organonitrogen compound biosynthetic process”, “tRNA aminoacylation”, “translation”, “peptide metabolic process”, “peptide biosynthetic process”, “amide acid activation”, and “amide biosynthetic process”, were enriched in proteins that only responded to blue light (Figure 4C). This result was consistent with our finding that moso bamboo seedlings had the highest protein content under blue light (Figure S4B). GO terms associated with sugar synthesis and metabolism, including “polysaccharide catabolic process”, “glucosamine-containing compound catabolic process”, “chitin metabolic process”, “chitin catabolic process”, “carbohydrate catabolic process”, “aminoglycan catabolic process”, and “amino sugar catabolic process”, were enriched in proteins responding only to red light (red light-specific responsive proteins) (Figure 4D). Interestingly, the “cell wall macromolecule catabolic process” was significantly enriched in the red light-specific responsive proteins. The above study showed that the root length, stem length, internode length and first internode length of moso bamboo seedlings under red light were all significantly longer than the height of moso bamboo seedlings under blue light (Figure 1A–G). Phytohormones are known to play important roles as key compounds in plant growth and development [28,29]. However, we did not obtain enrichment for hormone-related molecular processes. To find the reason, we counted the number of identified proteins and response proteins of different hormones. The results showed that our proteome contained very few phytohormone-associated proteins. In addition to 1.3% of auxin-related proteins identified, less than 1% of other hormone-related proteins and even fewer hormone-related light-responsive proteins were identified (Figure S4C). Therefore, we did not enrich for hormone-related biological processes. Further analysis of hormone-related proteins that individually respond to red light showed an up-regulation of an auxin transporter protein, ABCB19 (ATP-BINDING CASSETTE B19) [30] (PH02Gene02211), under red light (Figure S4D). We speculate that red light regulates the growth of moso bamboo through this protein.

### 2.5. Effects of Blue and Red Light on Photosynthesis in Young Moso Bamboo Seedlings

**Figure 5:**
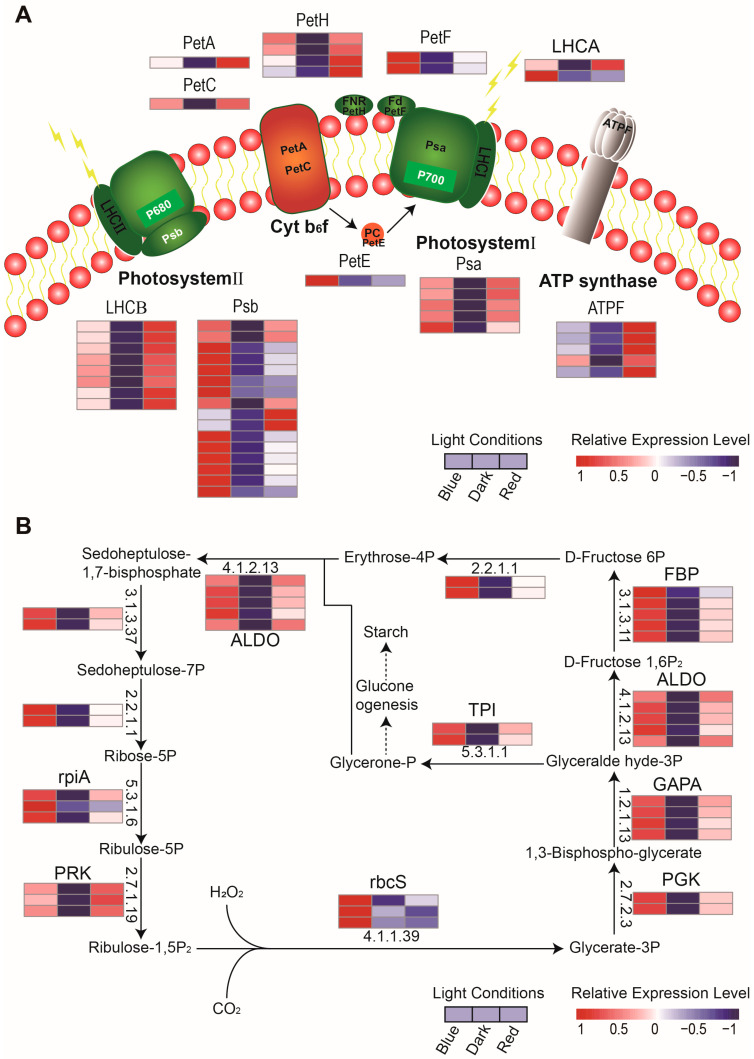
Differences in the regulation of photosynthesis-related proteins in moso bamboo seedlings under blue and red light. (**A**) Expression differences in the photosynthesis KEGG pathway-related DEPs in moso bamboo seedlings under different light treatments. (**B**) Expression differences in the carbon fixation in photosynthetic organism pathway-related DEPs under different light treatments. The arrows indicate the direction of substrate transformation. Heat maps were used to indicate the expression levels of the DEGs encoding the enzymes. (EC:4.1.1.39), ribulose-bisphosphate carboxylase large chain; (EC:2.7.2.3), phosphoglycerate kinase; (EC:1.2.1.13), glyceraldehyde-3-phosphate dehydrogenase (NADP+); (EC:4.1.2.13), fructose-bisphosphate aldolase, class I; (EC:3.1.3.11), fructose-1,6-bisphosphatase I; (EC:2.2.1.1), transketolase; (EC:3.1.3.37), sedoheptulose-1,7-bisphosphatase; (EC:5.3.1.6), ribose 5-phosphate isomerase A; (EC:2.7.1.19), phosphoribulokinase; (EC:5.3.1.1), triosephosphate isomerase (TIM).

Photosynthesis was significantly enriched in the enrichment analysis of blue and red light co-responsive proteins (Figure 4B). Furthermore, it was observed that more LHCA proteins were expressed in moso bamboo grown in blue light and more LHCB proteins were expressed in moso bamboo grown in red light (Figure 3D). These results indicate that although both red and blue light can regulate the expression of photosynthesis photosystem antenna proteins of moso bamboo, the patterns of regulating the expression of these proteins are different. To further investigate the effects of blue and red light on photosynthesis in moso bamboo seedlings, we analyzed the expression patterns of responsive proteins under different light conditions. We found that both blue and red light significantly up-regulated proteins in the photosynthesis-light and photosynthesis-dark reaction pathways compared to darkness (Figure 5A,B and Table S3). Therefore, we only analyzed the expression differences of photosynthesis-related proteins in bamboo under red and blue light. Unpaired Student’s *t*-test was used to compare the expression of these proteins under red and blue light. Fold change (Red/Blue) >2 or <0.5 and *p*-value < 0.05 defined differentially expressed proteins under red and blue light. We found that some key proteins for the photosynthetic light response were not significantly different in expression under blue and red light. These included the Psa (PHOTOSYSTEM I SUBUNIT) protein of photosystem I (PS I), the LHCB protein of photosystem II (PS II), the PetA (PHOTOSYNTHETIC ELECTRON TRANSFER A) and PetC (PHOTOSYNTHETIC ELECTRON TRANSFER C) proteins of the Cyt b6f complex, the PetF (PHOTOSYNTHETIC ELECTRON TRANSFER F) protein of the Fd electron transporter, and the PetH (PHOTOSYNTHETIC ELECTRON TRANSFER H) protein of the FNR (ferredoxin NADP + oxidoreductase) electron transporter (Figure S5A). Additionally, an LHCA protein in photosystem I was significantly more expressed under blue light than under red light. This result is consistent with the results shown in Figure 3D. This suggests that although some LHCA proteins in moso bamboo are up-regulated under red light compared to darkness, they are still less expressed than under blue light. Additionally, most of the Psb proteins in photosystem II were found to be more highly expressed under blue light, such as the PsbP, PsbR and PsbQ proteins, with the exception of the PsbS (PHOTOSYSTEM II SUBUNIT) protein, which was more highly expressed under red light. Furthermore, the three proteins of the F-type ATPase (ATPF) complex were all significantly more highly expressed under red light than under blue light. In the photosynthesis-dark reaction, only the rbcS protein, which catalyzes CO_2_ fixation to glycerate-3P, was found to be significantly more highly expressed under blue light than under red light (Figure S5B).

**Figure 6:**
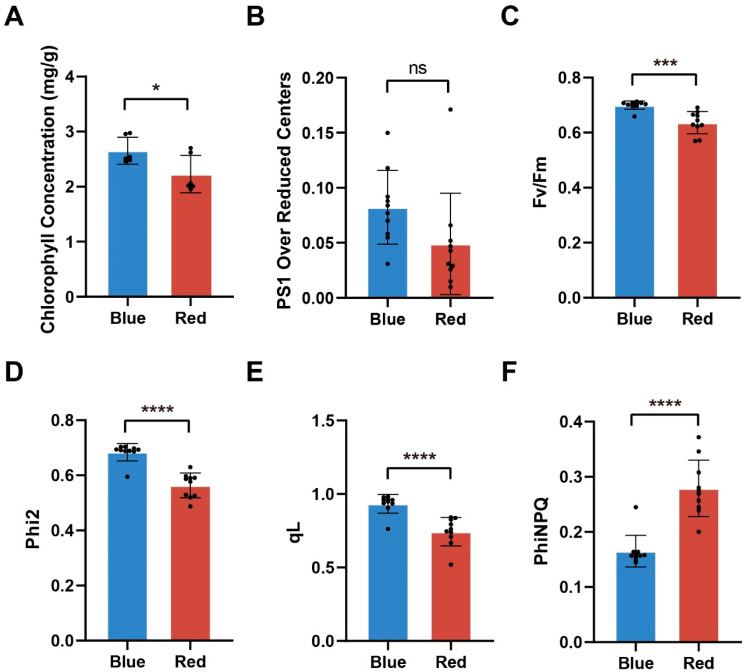
Comparison of chlorophyll content and fluorescence kinetic parameters of moso bamboo seedlings under red and blue light. (**A**–**F**) Chlorophyll concentrations (**A**), PS1 over reduced centers (**B**), Fv/Fm (**C**), Phi2 (**D**), qL (**E**) and PhiNPQ (**F**) for moso bamboo seedlings under different light treatments. Data are presented as mean ± SD (*n* = 10 biological replicates). Significant differences were analyzed by two-tailed Student’s *t*-test (^ns^ *p* > 0.05, * *p* < 0.05, *** *p* < 0.001, **** *p* < 0.0001).

Since moso bamboo does not perform photosynthesis in darkness, we only compared the chlorophyll content and chlorophyll fluorescence parameters of moso bamboo grown under red and blue light. We further compared the chlorophyll content and fluorescence kinetic parameters of moso bamboo seedlings under blue and red light. The results showed that the chlorophyll concentration of moso bamboo seedlings under blue light was significantly higher than that under red light (Figure 6A). In addition, chlorophyll a and chlorophyll b concentrations were significantly higher in moso bamboo seedlings under blue light than under red light (Figure S6A,B). This indicates that blue light has a greater effect on the synthesis of chlorophyll, chlorophyll a and chlorophyll b. Chlorophyll captures light and has an effect on the photosynthetic capacity of both PS I and PS II. Therefore, we measured chlorophyll fluorescence kinetic parameters of moso bamboo seedlings under different light conditions using the MultispeQ system, including PS1 over reduced centers (Figure 6B), PS I active centers (Figure S6C), maximum quantum efficiency (Fv/Fm) (Figure 6C), actual quantum efficiency (Phi2) (Figure 6D), quantum yield of photochemical quenching (qL) (Figure 6E), and quantum yield of regulatory energy dissipation (PhiNPQ) (Figure 6F). The measurements showed that PS I over reduced centers was significantly higher in blue light than in red light, indicating that PS I is more efficiently utilized in blue light than in red light. However, PS I active centers did not differ between blue and red light, indicating that there is no difference in electron reception/transfer by active PS I under different light conditions. Both Fv/Fm and Phi2 were significantly higher in blue light than in red light, indicating that blue light has a more effective potential maximum light energy conversion efficiency and actual light energy conversion efficiency. The lower qL content in red light compared to blue light indicates that PS II is less open in red light. The lower content of PhiNPQ in blue light compared to red light indicates that blue light has a lower proportion of non-photochemical quenching than red light and has a higher photochemical utilization efficiency. We speculate that more PsbR, PsbQ and PsbP proteins expressed in blue light compared to red light made the PS II of moso bamboo more open and had a higher photochemical utilization efficiency.

### 2.6. Effects of Blue and Red Light on the Metabolic Pathways of Soluble Sugars and Starch in Moso Bamboo Seedlings

**Figure 7:**
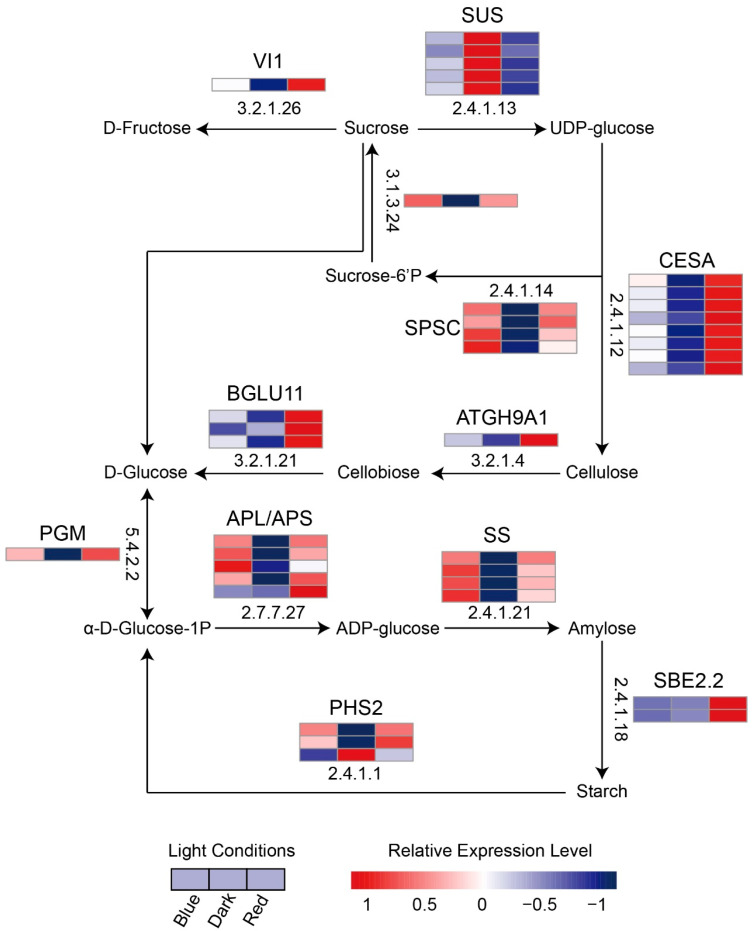
Expression differences in the starch and sucrose metabolic KEGG pathway-related DEPs of moso bamboo seedlings under different light treatments. The arrows indicate the direction of substrate transformation. The numbers in the boxes represent the EC numbers of the enzymes involved in the reactions. Heat maps were used to indicate the expression levels of the DEPs encoding the enzymes. (EC:3.2.1.26), beta-fructofuranosidase; (EC:2.4.1.13), sucrose synthase; (EC:2.4.1.12), cellulose synthase (UDP-forming); (EC:3.2.1.4), endoglucanase; (EC:3.2.1.21), beta-glucosidase; (EC:5.4.2.2), phosphoglucomutase; (EC:2.7.7.27), glucose-1-phosphate adenylyltransferase; (EC:2.4.1.21), starch synthase; (EC:2.4.1.18), 1,4-alpha-glucan branching enzyme; (EC:2.4.1.1), glycogen phosphorylase; (EC:2.4.1.14), sucrose-phosphate synthase; (EC:3.1.3.24), sucrose-6-phosphatase.

In the GO enrichment analysis of blue and red light co-responsive proteins and red light-specific responsive proteins, sugar and carbohydrate metabolism were significantly enriched (Figure 4B,D). As soluble sugars and starch play important roles in plant growth metabolism [31,32,33], we analyzed the expression patterns of light-responsive proteins related to sugar metabolism. Both blue and red light up-regulated the expression of light-responsive proteins in the soluble sugar and starch pathways compared to darkness (Figure 7 and Table S4). To compare the expression differences of these proteins under red and blue light more intuitively, we calculated the fold change of their expression levels between red and blue light conditions (Figure S7). We found that there were two cellulose synthetases CESA (CELLULOSE SYNTHASE) (PH02Gene24001 and PH02Gene34577), an enzyme ATGH9A1 (GLYCOSYL HYDROLASE 9A1) (PH02Gene12539) that directly degrades cellulose to cellobiose, and a protein BGLU11 (BETA GLUCOSIDASE 11) that catalyzes cellobiose to D-glucose. The expression level of these proteins was significantly higher under red light than under blue light. We speculate that the synthesis and degradation of cellulose is more active under red light than under blue light. In addition, we also found that although enzymes involved in the metabolism of glucose to starch such as PGM (PHOSPHOGLUCOMUTASE), APL (ADPGLC-PPASE LARGE SUBUNIT) (except for an APL2 protein that was more highly expressed in red light than in blue light and an APL1 protein that was more highly expressed in blue light than in red light), and SS (STARCH SYNTHASE) showed no significant difference in expression between red and blue light, SBE2. 2 (STARCH BRANCHING ENZYME 2.2), which converts amylose directly into starch, showed significantly higher expression in red light than in blue light. The expression of PHS2 (ALPHA-GLUCAN PHOSPHORYLASE 2), a direct starch degrading enzyme, was not significantly different between red and blue light. Based on these results, we speculate that starch accumulates in moso bamboo under red light. Although the expression of the sucrose catabolic enzyme SUS (SUCROSE SYNTHASE) protein was not significantly different between red and blue light, two SUS proteins had a fold change of less than 0.7 (0.66 and 0.63) (Table S4), so we speculate that red light may contribute to the accumulation of sucrose in moso bamboo.

**Figure 8:**
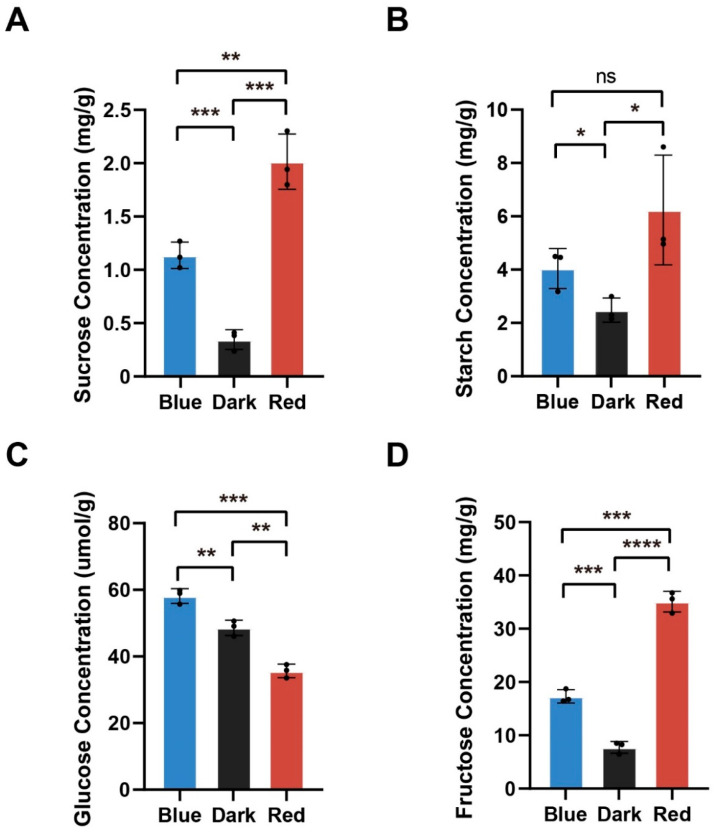
Comparison of soluble sugar and starch contents in moso bamboo seedlings under different light treatments. (**A**–**D**) Differences in sucrose (**A**), starch (**B**), fructose (**C**), and glucose (**D**) in moso bamboo under different light treatments. Data are presented as mean ± SD (*n* = 3 biological replicates). Significant differences were analyzed by two-tailed Student’s *t*-test (^ns^ *p* > 0.05, * *p* < 0.05, ** *p* < 0.01, *** *p* < 0.001, **** *p* < 0.0001).

We then compared the content of soluble sugars and starch in moso bamboo seedlings under blue light, red light and darkness. The results showed that the sucrose content was significantly higher under red light than under blue light and significantly higher under blue light than under darkness (Figure 8A). The amount of starch under both blue and red light was significantly higher than the amount of starch in the dark, and there was no significant difference in the amount of starch under blue and red light (Figure 8B). Glucose content was significantly higher under blue light than in the dark and significantly higher in the dark than under red light (Figure 8C). Fructose content was significantly higher under red light than under blue light and significantly higher under blue light than under darkness (Figure 8D). Although the glucose content of bamboo shoots under red light was the lowest compared to that under other treatments, the total soluble sugar content of bamboo shoots under red light was the highest (Figure S8). These results indicate that red light more strongly promotes the accumulation of sucrose, starch and fructose than blue light in moso bamboo. However, blue light more strongly promotes the deposition of glucose in moso bamboo.

### 2.7. Red Light Affects Cell Wall Synthesis of Moso Bamboo

**Figure 9:**
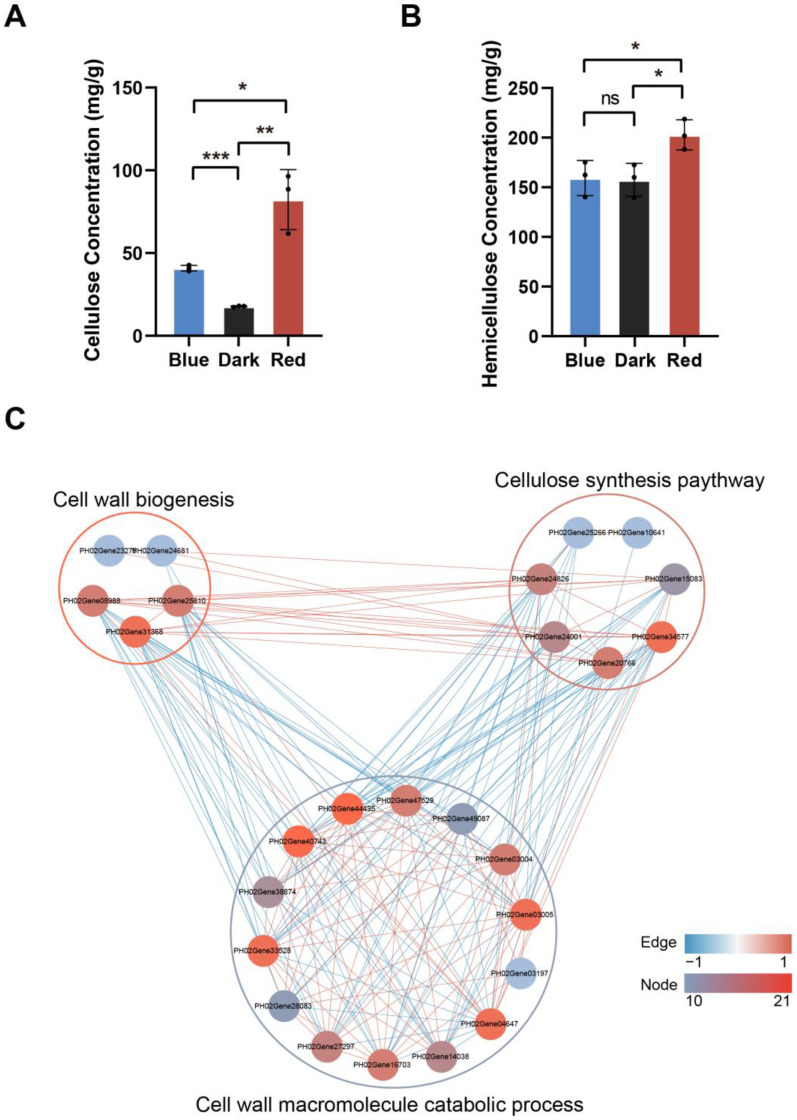
Compared to blue light, red light promotes the formation of cell wall components in moso bamboo seedlings. (**A**,**B**) Cellulose (**A**) and hemicellulose (**B**) contents of moso bamboo seedlings under different light conditions. Data are presented as mean ± SD (*n* = 3 biological replicates). Significant differences were analyzed by two-tailed Student’s *t*-test (^ns^ *p* > 0.05, * *p* < 0.05, ** *p* < 0.01, *** *p* < 0.001). (**C**) Correlation analysis of DEP expression levels between cell wall biogenesis, cell wall macromolecule catabolic process and cellulose synthesis pathways. The color of the node indicates the number of proteins with co-expression relationships (from left to right, the color bar indicates the number of proteins increases in turn), and the connecting lines indicate a significant co-expression relationship between the two connected proteins. The color of the line connecting two proteins is used to indicate the correlation between the proteins. The color of the line is between 0 and 1, indicating that there is a positive correlation between the two proteins. Contrarily, it shows that there is a negative correlation between the two proteins.

The cell wall macromolecule catabolic process was significantly enriched from red light-specific responsive proteins (Figure 4D). Moreover, red light-specific quantified proteins were mainly involved in the cell wall polysaccharide biosynthesis process (Figure 3B). The contents of the cellulose synthases and cellulose degrading enzymes in moso bamboo under red light were significantly higher than those in darkness and under blue light (Figure 7). Cellulose is the main component of plant cell walls. The above analysis suggests that red light plays an important role in the synthesis and degradation of moso bamboo cell wall. To further study the regulatory effect of red light on moso bamboo cell wall, we compared and analyzed the cellulose and hemicellulose content of moso bamboo seedlings under different light treatments. The results showed that the cellulose content of moso bamboo was significantly higher under red light than under blue light and significantly higher under blue light than under darkness (Figure 9A). The hemicellulose content of moso bamboo under red light was much higher than that under blue light and dark, whereas there was no difference between blue light and dark (Figure 9B). Then, we compared and analyzed the expression levels of cell wall biosynthesis-related proteins and cell wall macromolecule catabolism-related proteins under different light treatment conditions. The results showed that the expression of cell wall biosynthesis-related proteins, such as XTR3 (XYLOGLUCAN ENDOTRANSGLYCOSYLASE 3) (PH02Gene25810 and PH02Gene31368) and GXM1 (GLUCURONOXYLAN METHYLTRANSFERASE 1) (PH02Gene23275 and PH02Gene30719) proteins, increased (Figure S9A and Table S5), and the expression of cell wall macromolecular catabolism-related proteins (Figure S9B and Table S6), such as CHIV (CHINASE CLASS IV) (PH02Gene03197, PH02Gene14038, PH02Gene28083 and PH02Gene40743) and CHI-B (BASIC CHINASE) (PH02Gene03004, PH02Gene03005, PH02Gene16703, PH02Gene27297, PH02Gene38035 and PH02Gene49087) proteins, decreased under red light, compared with that under dark and blue light. To further investigate the relationship between the expression patterns of cellulose synthetase and cell wall synthesis and degradation proteins under red light, we calculated the Pearson correlation coefficients of the DEP expression levels of moso bamboo seedlings under different light treatments using Cytoscape. The results showed that there were close correlations between the expression levels of cellulose synthesis-related proteins, cell wall biogenesis-related proteins and cell wall macromolecule catabolism-related proteins (Figure 9C). Cellulose synthesis-related protein and cell wall biogenesis-related protein showed a strong positive correlation, and cell wall macromolecule catabolism-related protein and other proteins showed a strong negative correlation. These results indicate that red light plays a crucial role in the synthesis and degradation of the cell wall of moso bamboo seedlings.

## 3. Discussion

Moso bamboo is of important economic, social and cultural value due to its characteristics of fast growth, high mechanical strength, and strong carbon fixation capacity [1]. Light is the important energy source and environmental signal of moso bamboo and also regulates moso bamboo growth and development [34,35,36]. It has been reported that blue and red light have various effects on plant growth and development [18]. Thus, further understanding the growth and development of moso bamboo seedlings regulated by blue and red light may improve cultivation of moso bamboo seedlings under various light conditions and pave the way for genetic breeding [37]. This study investigated the phenotypic differences of moso bamboo seedlings grown under blue light, red light and dark conditions and studied the proteomic changes in moso bamboo seedlings using proteomic methods.

From the phenotypic observation, we found that red light is more effective in promoting the horizontal and vertical growth of moso bamboo. Similarly to other plants under red light, such as rice [19], cucumber [38], and lettuce [39], bamboo under red light shows the phenomenon of “red light syndrome”, indicating the stem length is longer than that under blue light, and the chlorophyll content is also lower than that under blue light [40] (Figure 1C and Figure 6A). However, compared with blue light, red light promotes the dry weight accumulation of moso bamboo (Figure 1G), and the stem diameter is thicker (Figure S1A). These light phenotypes of moso bamboo are different from the phenomenon of biomass reduction in “red light syndrome” found in other plants, suggesting a specific light-regulation network in moso bamboo.

In order to explain this phenomenon, we carried out GO enrichment analysis on the specific quantitative proteins and light response-related proteins of moso bamboo seedlings under different light treatments and determined the biological processes in which these proteins are involved. We found that the expression of moso bamboo HY5 protein was significantly promoted by red light. HY5 is an important protein in plant photomorphogenesis, and light will promote the accumulation of HY5 protein and inhibit plant elongation [41,42]. Therefore, the stem length of moso bamboo under red light is longer than that under blue light, which is not due to the regulation of HY5 protein. In addition, the protein responding to red light does not enrich the protein related to photomorphogenesis, so we speculated that the regulation effect of red light on photomorphogenesis of moso bamboo would be different from that of other plant species. Although both red light and blue light regulate the light reaction and dark reaction of photosynthesis, we found that moso bamboo expressed more LHCB protein under red light and more LHCA protein under blue light (Figure 3D). Previous research has shown that blue light promotes the expression of the LHCB gene in peas [43]. Additionally, red light promotes the expression of LHCA and some LHCB in Zostera marina L, while blue light only promotes the expression of ZmLHCA6 [44]. This suggests that the types of LHC proteins promoted by red and blue light differ among different plant species. In addition to LHCB protein, moso bamboo will express more PsbP, PsbQ and PsbE proteins in photosystem II under blue light (Figure 5A and S5A). This may be the reason why moso bamboo photosystem II has higher efficiency of light energy conversion under blue light. It is consistent with the previous research finding that PsbP and PsbQ coordinate the donor and receptor activities of photosystem II and stabilize the activity of PSII-light-harvesting complex II (LHCII) [45].

Because the specific CO_2_ absorption efficiency of moso bamboo has not been measured, we could not assess the differences in fixed CO_2_ between the moso bamboo seedlings grown under red light and those grown under blue light. We measured the total soluble sugar content of moso bamboo under different light treatments and found that red light promoted sugar accumulation in moso bamboo more effectively (Figure S8). Then, we focused on the regulatory effect of red light on the cellulose content of moso bamboo. We found that moso bamboo under red light expressed more cellulose synthetase CESA and cellulose degrading enzyme ATGH9A1 (Figure 7 and Figure S7), and we also found that moso bamboo under red light had more cellulose content (Figure 9A). Cellulose is the main component of plant cell walls [46,47], and cell division and elongation of plants in the process of growth require loosening of the cell wall matrix and deposition of new cell wall components [48]. Previous studies have shown that the red light receptor PHYB (PHYTOCHROME B) in *Arabidopsis* regulates the synthesis of hypocotyl cellulose [49]. No significant difference was observed in the cellulose content of tomato seedlings under red light and blue light [50]. It can be speculated that there is a unique pathway in bamboo that enables it to produce cellulose more efficiently using red light. Red light promotes the height of sweet pepper plants, while blue light promotes the stem diameter of sweet pepper [51]. In addition, red light increases the diameter of the hypocotyl of soybean compared to darkness, but inhibits hypocotyl elongation [52]. In contrast, stem elongation of moso bamboo is the same length under red light and darkness. Previous studies have suggested that red light reduces the degradation of cell wall polysaccharides, making the cell wall more firm and inhibiting hypocotyl elongation [52]. This may explain the difference between moso bamboo and soybean under red light, as bamboo not only expresses more cellulose synthesis enzymes but also more cellulose degradation enzymes, which may provide a flexible environment for cell growth. In addition, compared with moso bamboo grown under blue light, the expression of cell wall synthetic protein was higher and the expression of cell wall macromolecule degradation protein was lower in moso bamboo under red light. In addition, red light also specifically promoted the expression of some proteins of the cell wall macromolecule biosynthesis process (Figure 3B and Figure S8). These results suggested that red light can promote the formation of cell walls in moso bamboo. Plant hormones are also essential for the elongation and division of plant cells. We found high expression of auxin transporter ABCB19 (Figure S4D) in moso bamboo under red light. *Arabidopsis* ABCB19 protein mutant seedlings showed hypersensitivity in inhibiting hypocotyl elongation, root tropism and lateral root growth under red light [53], implying that ABCB19 protein plays a key role in the regulation of stem elongation and lateral root growth of moso bamboo under red light.

In summary, similarly to other plants [19,54], moso bamboo grown under blue light has more chlorophyll and a higher rate of photosynthetic photoreaction than bamboo grown under red light. However, moso bamboo expresses more cellulose synthases and proteins in the cell wall biogenesis process and fewer proteins in the cell wall macromolecule catabolic process, which is different from other plants [50], and may result in the accumulation of more cellulose and higher levels of dry weight in moso bamboo. These findings pave the way for further determination of the optimal light conditions for the cultivation of moso bamboo seedlings and moso bamboo breeding.

## 4. Materials and Methods

### 4.1. Plant Materials and Treatments

After being shelled and cleaned with water twice, moso bamboo seeds were soaked in water for 48 h in the dark at 25 °C. Then, the soaked seeds were planted in soil and left to germinate in the dark at 25 °C. The germinated moso bamboo seedlings were transferred to 30 µmol m^−2^ s^−1^ red light (650 nm) or blue light (450 nm) or kept in the dark at 25 °C and 70% relative humidity for 14 days, and then were used for the observation of physiological phenotypes and statistical analysis of physiological indicators.

### 4.2. Protein Preparation and Digestion

Extraction of total protein from moso bamboo seedlings was based on previously described methods [55]. The seedlings of moso bamboo grown for 14 days under different light conditions were collected together and immediately frozen and ground into powder in liquid nitrogen. Three 0.5 g powder samples from each condition were weighed (3 repetitions), added to 2 mL lysis buffer (0.5 M Tris-HCl pH 8.0, 50 mM EDTA, 0.1% SDS, 0.1 M KCl, 0.7 M Sucrose, 50 mM Dithiothreitol (DTT), Protease inhibitor), mixed well, and then placed on ice for lysing for 30 min. The lysates were centrifuged at 12,000 rpm for 30 min at 4 °C, and phenol was added to the supernatants for extraction of whole protein. Six volumes of precooled 100% acetonitrile (ACN) were added to the supernatants, which were then were stored at −20 °C overnight. The protein precipitates were washed three times with precooled acetone and then concentrated in vacuum for 5 min. The protein was resuspended in solution buffer (8 M Urea, 0.1 M Tris-HCl pH 8.0). Protein concentration was determined with the Pierce BCA Protein Assay Kit (23,227, Thermo Fisher Scientific, Carlsbad, CA, USA).

A 500 μg quantity of dissolved protein was reduced by 50 mM DTT (3483-12-3, Sigma-Aldrich, Burlington, MA, USA) at 37 °C for 1 h. Then, 150 mM iodoacetamide (144-48-9, Sigma-Aldrich, MA, USA) was added to alkylate the reduced sulfyhdryl groups. After 30 min in the dark at room temperature, the protein was diluted by 4 volumes 0.1 M Tris-HCl (pH 8.0) and digested with trypsin (V5111, Promega, Madison, WI, USA). The digestive product was desalted with MonoSpin C18 spin column (GL Sciences, 5010-21701, Tokyo, Japan), eluted with 60% acetonitrile, and dried in rapid vacuum.

### 4.3. Data-Independent Acquisition Mass Spectrometry

The dried protein sample was resuspended with solvent A (A: water with 0.1% formic acid), and iRT peptides (ki-3002-2, Biognosys, Schlieren, Switzerland) were added. Mass spectrometry analysis was conducted by online nanospray LC-MS/MS on an Orbitrap Fusion Lumos coupled to an EASY nano LC system (Thermo Fisher Scientific, Carlsbad, CA, USA). During LC-MS/MS operation, 1 µg peptide sample was loaded into a trap column (Thermo Fisher Scientific Acclaim PepMap C18, 100 µm × 2 cm, CA, USA) with a flow rate of 10 µL/min; then, in the analytical column (Thermo Fisher Scientific Acclaim PepMap C18, 75 µm × 15 cm, CA, USA) with 3–32% solvent B (B: ACN with 0.1% formic acid), linear gradient was used to separate polypeptides for 120 min. The column flow rate was maintained at 300 nL/min. The electric spray voltage of Nanospray Flex ion source was 2.3 kV. Using full scan in Orbitrap, the AGC target was 8 × 10^4^, the max injection time was 120 ms with resolution of 60,000. Data-independent acquisition scanning was performed after each MS scan. The AGC target was 4 × 10^5^, and the max injection time was 22 ms. A total of 42 variable DIA windows were set, and the acquisition range was from *m*/*z* 350 to *m*/*z* 1500 with resolution of 30,000. The sample was fragmented by higher energy collision-induced dispersion (HCD) with 32% collision energy.

### 4.4. Data Analysis

Before signal extraction and quantification, the “DirectDIA” mode of Spectronaut 16 (Biognosys AG, Schlieren, Switzerland) was used to generate a DIA spectrum library using the raw data described previously [55] (http://forestry.fafu.edu.cn/pub/cells, accessed on 10 October 2022) and the moso bamboo protein FASTA file (http://forestry.fafu.edu.cn/db/PhePacBio/download.php, accessed on 10 October 2022). The DIA raw file was directly analyzed using the default parameters. A maximum of two missed cleavages were allowed. Carbamidomethyl (C) was set as fixed decoration, and Occupation (M) and Acetyl (Protein N-term) were set as variable decoration. FDR at peptide and protein levels was set at 1%. The retention time was corrected with iRT peptide. After unpaired Student’s *t* test, proteins with the expression fold change (FC) ratio > 2 or <0.5 and *p*-value < 0.01 were filtered and defined as DEPs.

### 4.5. Bioinformatic Analysis

Blast2GO software (v6.0.3, Valencia, Spain) was used to annotate the function of bamboo protein database and used for the functional enrichment analyses of DEPs [56]. Single-tailed Fisher’s exact test was used for the functional enrichment, and GO terms with FDR < 0.05 were screened out. Biological process (BP) was selected to display. Interactive Graph visualized GO items with FDR < 0.05 through REVIGO [57]. Protein fasta sequences were used for KEGG annotation of DEPs through an online website (https://www.kegg.jp/blastkoala/, accessed on 25 October 2022) [58]. A Venn diagram was generated using Venn Diagram (v1.7.3) in R. The heat maps were generated using the pheatmap package (v1.0.12) in R. The principal component analysis (PCA) was generated using FactoMineR (v2.6) in R.

### 4.6. Measurement of Chlorophyll Content and Chlorophyll Fluorescence Parameters

The seedlings of moso bamboo grown for 14 days under different light conditions were collected and ground in liquid nitrogen. A 0.1 g amount of powder was weighed and added to 10 mL abstracting solution (anethanol:acetone:distilled water = 4.5:4.5:1, *v*/*v*/*v*). Then, the tube was wrapped in aluminum foil and placed in the dark for 3 days until the sample turned white. After centrifugation, the supernatant was used to measure OD at 645 nm and 663 nm with a spectrometer. The chlorophyll content was calculated using the following equation [59]: Chlorophyll (mg/g) = (8.02 × OD663 + 20.20 × OD645) × V/W, Chlorophyll a (mg/g) = (12.72 × OD663 − 2.69 × OD645) × V/W, Chlorophyll b (mg/g) = (22.88 × OD645 − 4.68 × OD663) × V/W, where V is the volume of the extract (in milliliters) and W is the fresh weight of the sample powder (in milligrams).

A MultispeQ system (MultispeQ V2, MI, USA) was used to measure chlorophyll fluorescence parameters of plants under different lighting conditions. For each treatment, the second leaves of 10 moso bamboo seedlings were measured. The parameters were calculated by the instrument using the following formulas and principles:

(1) The total active PSI centers in the leaves under a certain condition are proportional to PM, the maximal absorption difference between dark and the second saturation pulse taken after application of far red light to oxidize electron carriers [60].
(2) PSI over-reduced (PSIor) can be estimated from the difference in saturating pulse-induced absorbance changes taken in steady state and after far red illumination [60].
(3) Fv/Fm = (Fm − Fo)/Fm [61]
(4) Phi2 = (F’m − Fs)/F’m [61]
(5) qL = (F’m − Fs)/(F’m − F’o) × (F’o/Fs) [62]
(6) PhiNPQ = 1 − (F’m − Fs)/F’m − Fs/Fm [63]

Fm is the value of maximal fluorescence after dark adaptation. Fo is the value of minimal fluorescence after dark adaptation. F’m is the value of maximal fluorescence in the light-adapted state. Fs is the relative steady-state fluorescence yield. F’o is the value of minimal fluorescence in the light-adapted state.

### 4.7. Measurement of Starch, Sucrose, Fructose, Glucose, Total Soluble Sugar, Cellulose and Hemicellulose Content

A 0.1 g amount of moso bamboo powder was weighed for the following analyses. The starch levels were determined by Amylum Content Assay Kit (Sangon Biotech, Shanghai, China, D799325-0050). The sucrose levels were determined by Plant Sucrose Content Assay Kit (Sangon Biotech, Shanghai, China, D799789-0050). The glucose levels were determined by Glucose Content Assay Kit (Sangon Biotech, Shanghai, China, D799407-0050). The fructose levels were determined by Plant Tissue Fructose Content Assay Kit (Sangon Biotech, Shanghai, China, D799403-0050). The extraction of soluble sugars from bamboo shoots was based on a previously reported method with slight modifications [64]. A 0.1 g amount of bamboo shoot powder was weighed and added to 5 mL of distilled water. The mixture was heated in boiling water for 30 min, then centrifuged to transfer the supernatant to a new tube. After repeating the process once, 0.5 mL of the supernatant was taken and mixed with 1 mL of 9% phenol and 5 mL of concentrated sulfuric acid. After shaking well, the mixture was left to stand at room temperature for 30 min, and the absorbance at 485 nm was measured. The soluble sugar content in the bamboo shoot sample was quantified by generating a standard curve for sucrose. To determine the cellulose and hemicellulose contents, 0.3 g of bamboo powder ground in liquid nitrogen was weighed, and then the cellulose levels were measured by Cellulose Content Assay Kit (Sangon Biotech, Shanghai, China, D799127-0050). A 0.05 g amount of dried and fully ground bamboo powder from the samples under different light treatments was weighed. Hemicellulose Content Assay Kit (Sangon Biotech, Shanghai, China, D799022-0050) was used to measure the hemicellulose contents.

### 4.8. DEP Correlation Analysis and Network Construction

Expression Correlation (v1.1.0) in Cytoscape (v3.9.1, Bethesda, MD, USA) [65] was used to calculate the Pearson correlation coefficient according to the protein intensity values of DEPs in cellulose synthesis pathway, cell wall biology and cell wall macromolecule catalytic process under different lighting conditions, and the correlation network was constructed. The gene network was used for the default cutoffs “−0.95 and 0.95”.

### 4.9. Statistical Analysis

All data were analyzed with GraphPad Prism (v 8.3.0), and significant differences were analyzed with unpaired two-sided Student’s *t*-test. The significant levels were: ^ns^ *p* > 0.05, * *p* < 0.05, ** *p* < 0.01, *** *p* < 0.001, **** *p* < 0.0001.

## Data Availability

The mass spectrometry proteomics data have been deposited with the ProteomeXchange Consortium via the PRIDE [66] partner repository under the dataset identifier PXD039570.

## Supplementary Materials

The following supporting information can be downloaded at: https://www.mdpi.com/article/10.3390/ijms24065103/s1.
