# Supplementary material for: Data-Independent Acquisition Proteomics Reveals the Effects of Red and Blue Light on the Growth and Development of Moso Bamboo (Phyllostachys edulis) Seedlings
Source: Int J Mol Sci. 2023 Mar 7;24(6):5103. doi: 10.3390/ijms24065103 (PMC10049362; doi:10.3390/ijms24065103)
Supplement: Supplementary file 1 [file ijms-24-05103-s001.zip › ijms-2258961-supplementary.pdf]

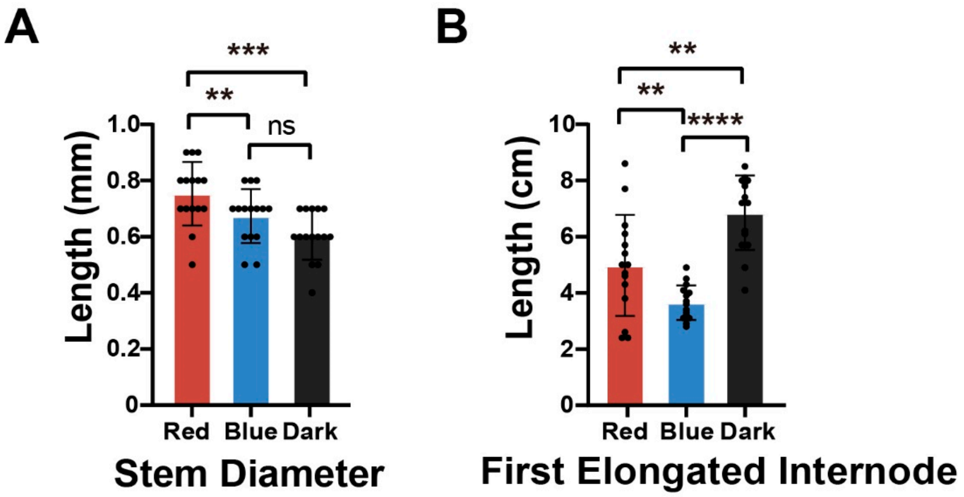

Figure S1. (A-B) Statistical analysis of the thickness of the stem diameter (A) and length of the first elongated internode (B) of moso bamboo seedlings under different light treatments. Data are presented as mean ± SD (n = 15 seedlings). Significant differences were analyzed by two-tailed Student's t-test (^{ns} $p > 0.05$, * $p < 0.05$, ** $p < 0.01$, *** $p < 0.001$, **** $p < 0.0001$).

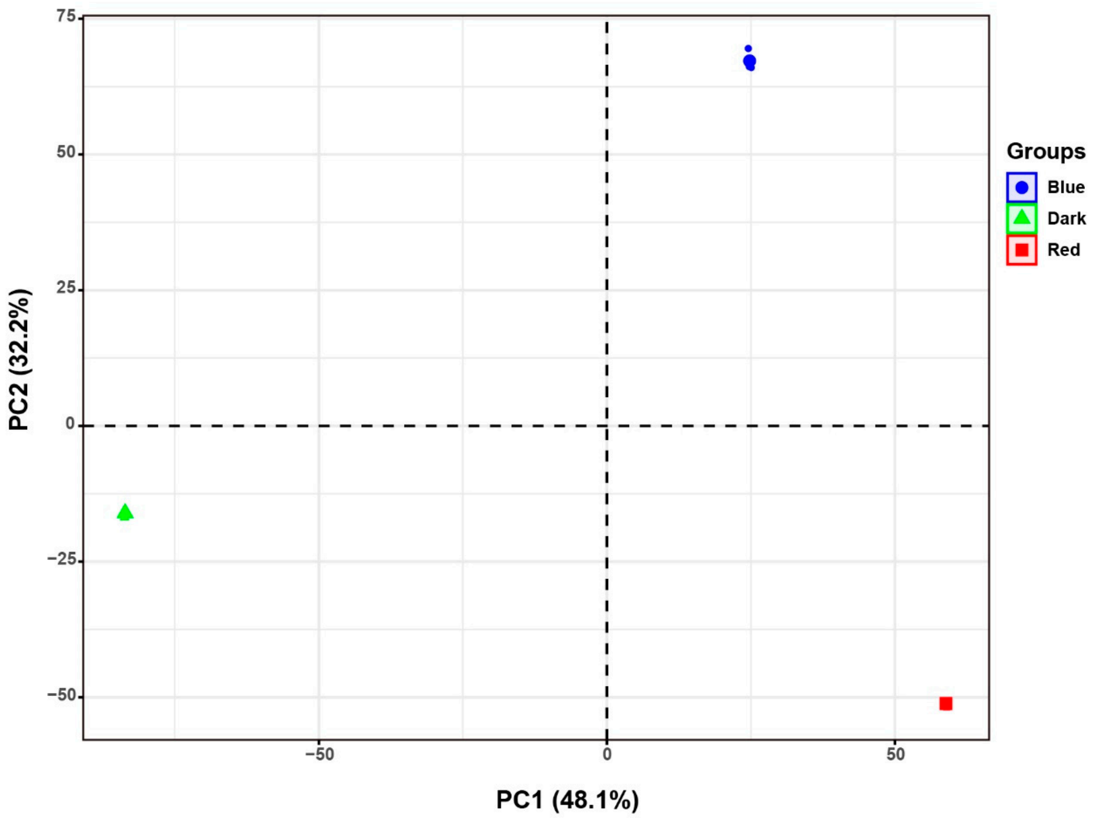

Figure S2. Principal component analysis (PCA) of the shared quantified proteins of the samples under different light treatments.

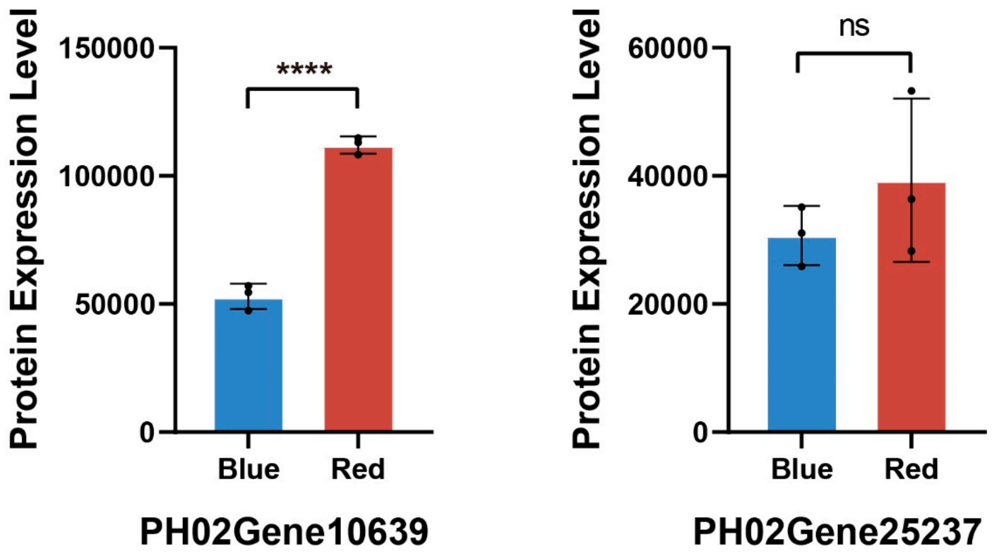

Figure S3. Comparison of the protein expression levels of two HY5 proteins only quantified in moso bamboo seedlings under red and blue light conditions. Data are presented as mean ± SD (n = 3 biological replicates). Significant differences were analyzed by two-tailed Student's t-test (^{ns} $p > 0.05$, **** $p < 0.0001$).

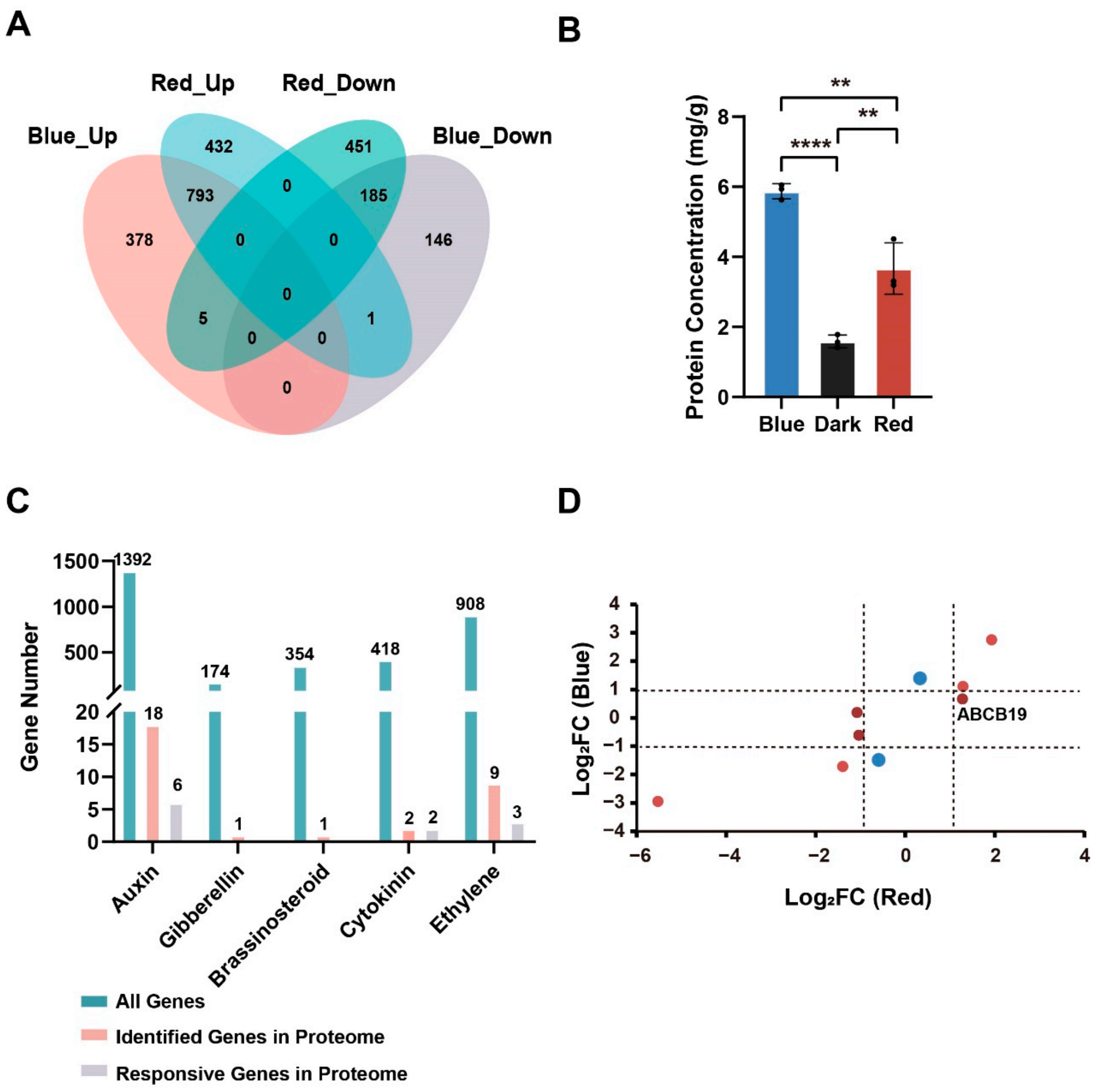

Figure S4. **(A)** Venn diagram showing the overlap of the DEPs in moso bamboo under different light conditions. **(B)** Comparison of the protein content extracted from moso bamboo grown under different light treatment conditions. Data are presented as mean ± SD (n = 3 biological replicates). Significant differences were analyzed by two-tailed Student's t-test (** $p < 0.01$, **** $p < 0.0001$). **(C)** Bar graph showing the number of identified phytohormones and the number of phytohormones in response to light in moso bamboo. **(D)** Scatterplot showing the association between the blue light-responsive proteins and red light-responsive proteins of phytohormones in moso bamboo. The dash lines indicate Log2 (FC) = ±1.

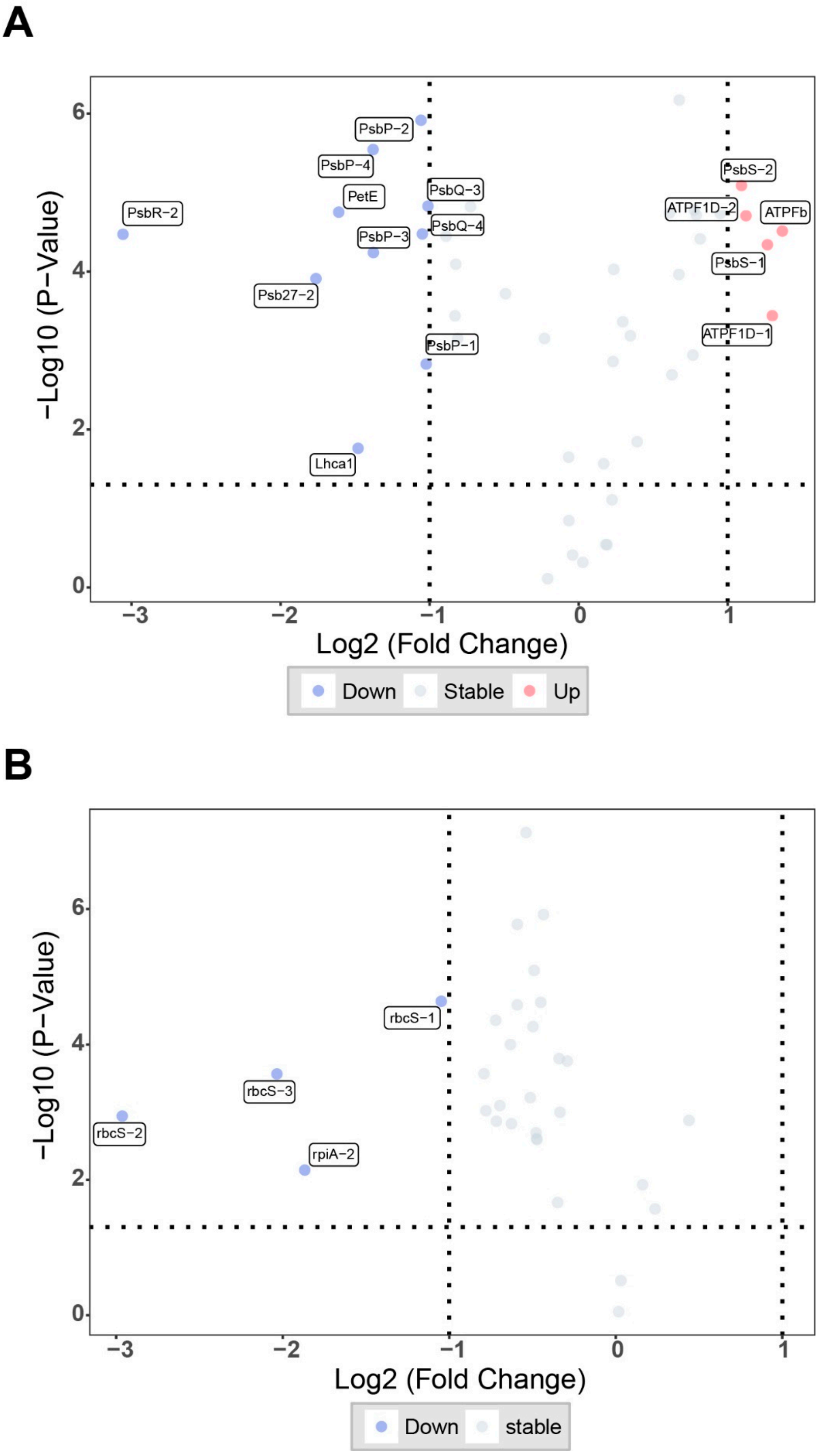

Figure S5. (A) Scatter plot showing differentially expressed proteins in response to red and blue light in the photosynthetic photoreaction of moso bamboo. **(B)** Scatter plot showing differentially expressed proteins in response to red and blue light in the photosynthetic dark reaction of moso bamboo. The dash lines indicate Log2 (FC) = ±1 and −Log10 (P-Value) = 0.05.

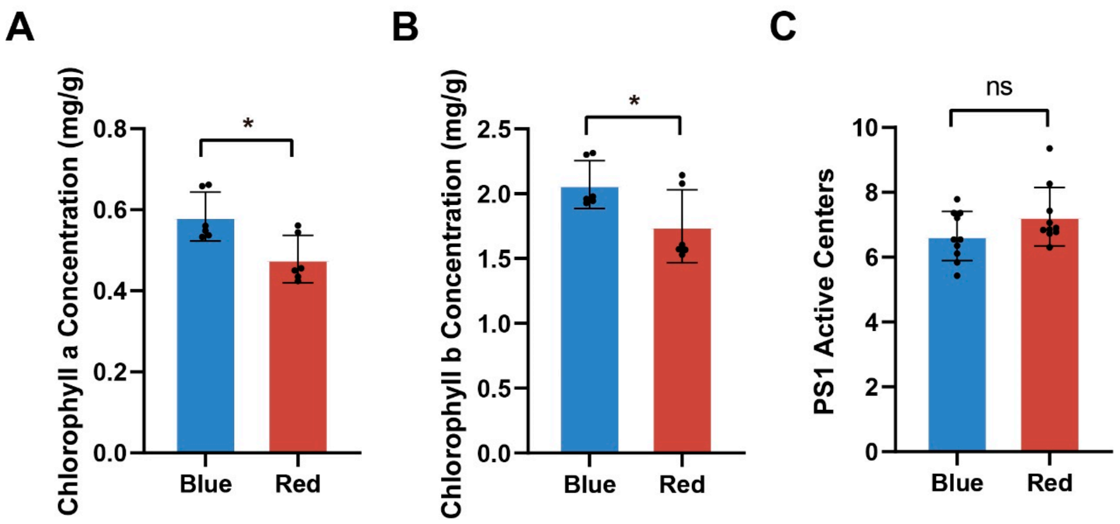

Figure S6. Comparison of chlorophyll content and fluorescence kinetic parameters of moso bamboo seedlings under red and blue light. **（A-C）** Chlorophyll a concentrations (A), Chlorophyll b concentrations (B), PS1 active centers (C) for moso bamboos seedlings under different light treatments. Data are presented as mean ± SD (*n* = 10 biological replicates). Significant differences were analyzed by two-tailed Student's *t*-test (^{ns} $p > 0.05$, * $p < 0.05$).

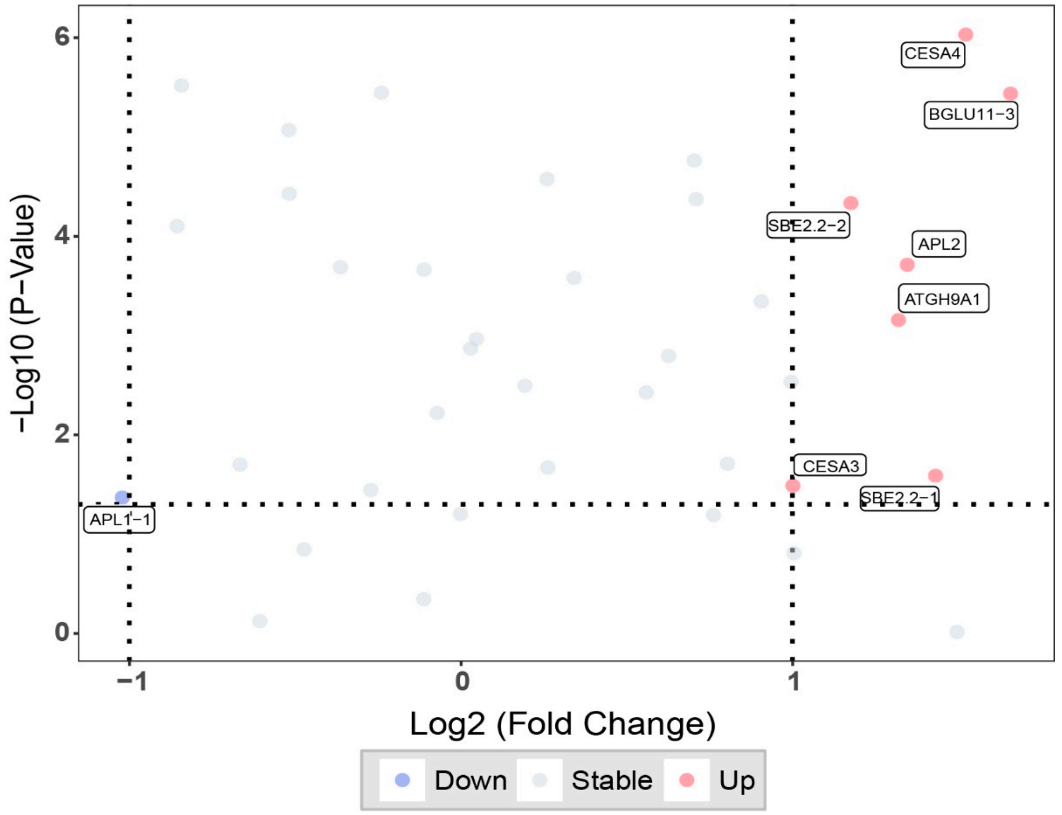

Figure S7. Scatter plot showing differentially expressed proteins in response to red and blue light in the starch and sucrose metabolic pathways of moso bamboo. The dash lines indicate Log2 (FC) = ±1 and −Log10 (P-Value) = 0.05.

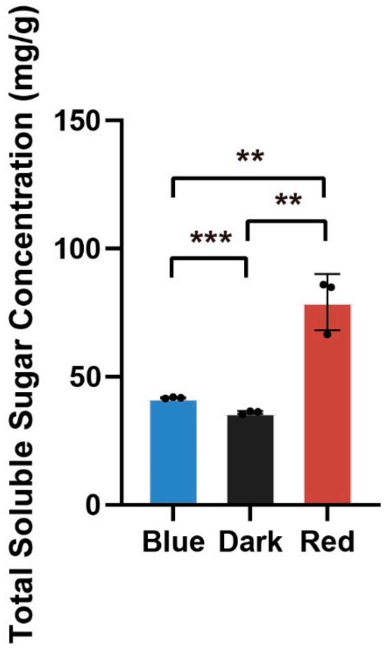

Figure S8. Comparison of total soluble sugar content in moso bamboo seedlings under different light treatments. Data are presented as mean ± SD (n = 3 biological replicates). Significant differences were analyzed by two-tailed Student's t-test (** $p < 0.01$, *** $p < 0.001$).

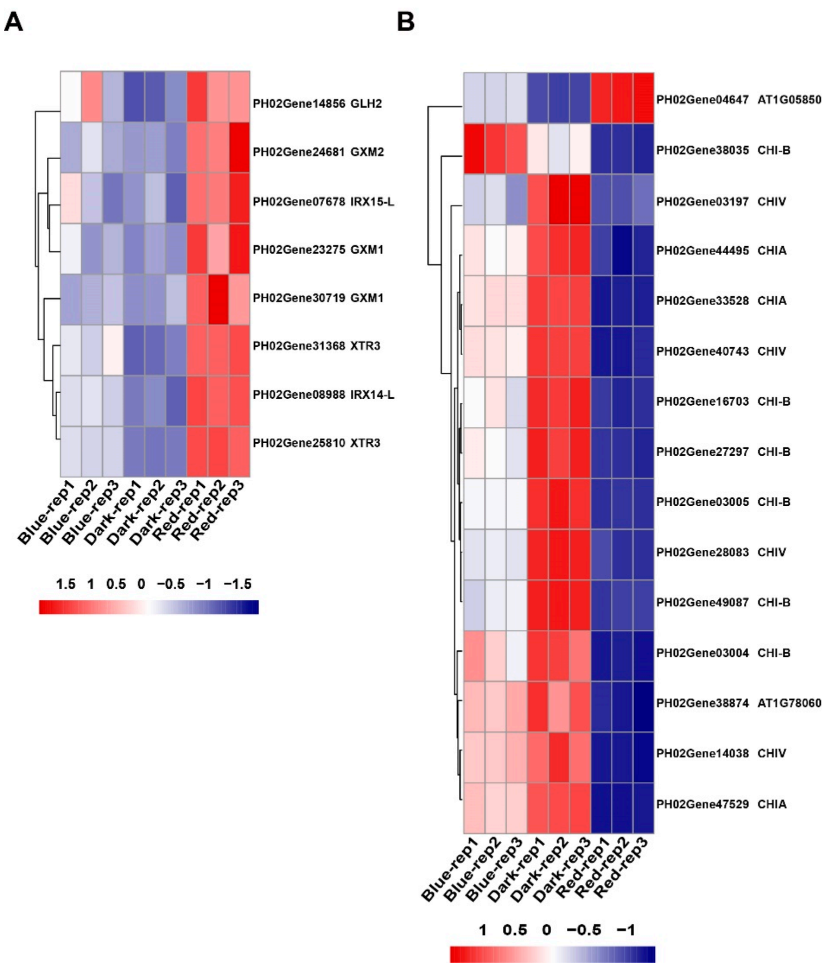

Figure S9. Expression profiles of DEPs involved in cell well biogenesis and cell wall

macromolecule catabolic process. (A-B) Heatmap showing the expression levels of cell well biogenesis-related proteins (A) and cell wall macromolecule catabolic process-related proteins (B) in moso bamboo seedlings under different light treatments. The numbers above the color bars refer to the detailed variation in expressed levels of the proteins.
